# Decoding gene essentiality in *Streptococcus suis* using Tn-seq and genome-scale metabolic modeling

**DOI:** 10.1128/spectrum.01303-25

**Published:** 2025-08-14

**Authors:** Yaoqin Hong

**Affiliations:** 1 Biomedical Sciences and Molecular Biology, College of Medicine and Dentistry, James Cook University 104560, Townsville, Queensland, Australia; 2 Australian Institute of Tropical Health and Medicine, James Cook University 104403, Townsville, Queensland, Australia; Institute of Microbiology, Chinese Academy of Sciences, Beijing, China

**Keywords:** gene essentiality, transposon sequencing, genome-scale metabolic modeling, *Streptococcus suis*

## Abstract

High-throughput transposon mutagenesis methods, such as transposon sequencing, are powerful tools for genome-wide identification of essential and conditionally essential genes in bacterial pathogens. In a recent study, Y. Zhang, R.Gong, M.Liang, L.Zhang, et al. (Microbiol Spectr 13:e0279124, 2025, https://doi.org/10.1128/spectrum.02791-24) applied Himar1-based Tn-seq to *Streptococcus suis*, generating a relatively dense mutant library, in combination with genome-scale metabolic modeling, to identify 244 candidate essential genes. Aside from the well-characterized antibiotic targets, there are several novel candidates currently being explored for drug development against other critical pathogens, and a number of previously uncharacterized potential targets were uncovered in classical model organisms. The study highlights the value of high-throughput transposon mutagenesis and genome-scale metabolic modeling in a less-characterized zoonotic pathogen and contributes important genetic insights that may inform future antimicrobial strategies.

## COMMENTARY

The application of transposons, or “jumping genes,” as a forward genetic tool originated in the 1970s with the use of Tn5 to induce random insertional mutations in bacterial genomes, facilitating the identification of genes involved in key metabolic pathways (1). Early breakthroughs, such as linkage mapping and inverse polymerase chain reaction, were instrumental in connecting phenotypes to underlying genetic determinants (2). These pioneering approaches laid the foundation for the sophisticated, high-throughput systems that define modern transposon mutagenesis (2).

With the advent of next-generation sequencing (NGS) technologies, the field of functional genomics has witnessed rapid advancement, enabling genome-wide assessments of gene essentiality and function at unprecedented resolution. Multiple methodologies, such as Tn-seq, TraDIS, INSeq, and HITS, have since emerged, combining dense transposon mutant libraries with NGS to assess gene function and essentiality genome-wide (2, 3). These systems, while conceptually similar, differ in the design of their transposon constructs, sequencing strategies, and downstream bioinformatics, each presenting distinct advantages and limitations depending on the target organism and research question (2, 3).

*Streptococcus suis* is a major cause of severe diseases in pigs, e.g., meningitis and septicemia (4). It also represents a zoonotic hazard, with human infections transmitted primarily through occupational or foodborne exposure (4). A study in Thailand estimated that *S. suis* infections in humans result in an annual economic loss of approximately US$11.3 million (5). As with many bacterial pathogens, the alarming rise in antimicrobial resistance in *S. suis* complicates treatment, and this highlights the urgency of identifying novel druggable targets (4).

In a recent study, Zhang et al. (6) employed the Himar1 Tn-Seq, a well-established tool for insertional mutagenesis, to define gene essentiality in this relatively understudied bacterium (7). Using this approach, Zhang et al. (6) reported a robust library of 150 essential genes in *S. suis*. In this study, it should be noted that mutants with slower growth are particularly vulnerable to being outcompeted by those retaining a wild-type-like growth rate due to the recovery method used. Assuming equal starting frequencies and exponential growth, the proportion (*P*) of slow-growing cells at time *t,* or *P*_slow_(*t*), can be modeled by the following simplified logistic function


$$P_{slow}\left(t\right)=\;\;\frac{1}{1+e^{\left(G_{normal}-G_{slow}\right)\;\times \;t}\;},$$

where *G* represents growth rate. A mutant with a 60 minute doubling time competing against a 30 minute doubling strain would theoretically decline to approximately 1.5% within 6 hours, although actual dynamics may vary depending on culture density, batch culture effects, nutrient depletion, and stochastic fluctuations. Further prolonging the recovery period would exacerbate this exclusion, leading to overestimation of essentiality.

Zhang et al. (6) used an extended 12 hour competitive recovery phase for enrichment of the mutant pool, followed by plating and selection of 100 colonies from each plate for downstream processing. Although this approach was intended to ensure adequate mutant enrichment, this extended recovery phase likely introduces competitive bias by favoring normal-growth clones and the quick displacement of the slow-growth mutant pool. An independent study addressed this limitation by using only a 2 hour post-electroporation recovery prior to low-density plating on solid media, in which the whole plate was harvested for TraDIS and the determination of essential genes (8). Such a strategy reduces competitive exclusion, as it allows slow-forming colonies to emerge without direct competition, thereby ensuring a more accurate assessment of essentiality by forward genetics.

**Fig 1:**
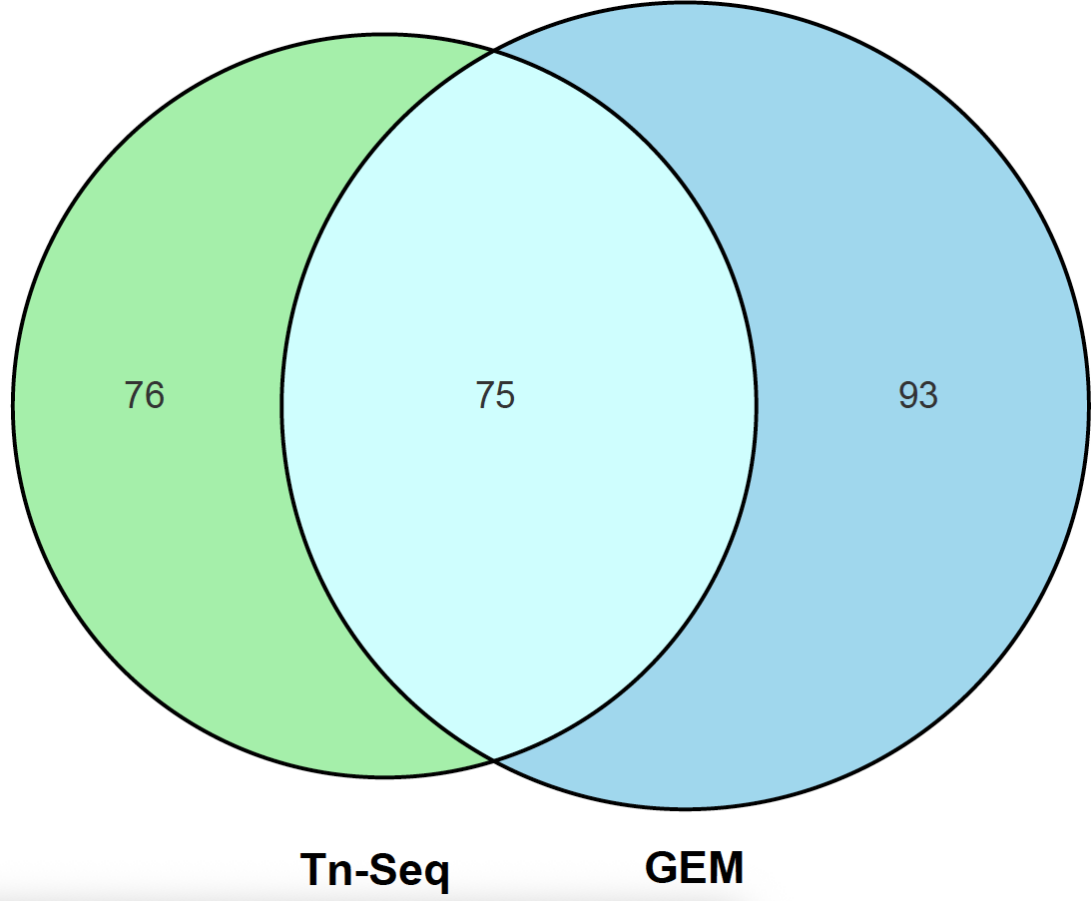
Venn diagram compares Tn Seq and GEM candidate essential gene datasets from Zhang et al. (6). Tn Seq contains 76 unique entries, GEM contains 93 unique entries, and 75 entries are shared between both, highlighting partial overlap in data.

Genome-scale metabolic modeling (GEM) offers a powerful framework to reconstruct and analyze the full complement of metabolic reactions encoded by an organism’s genome. In the study by Zhang et al. (6), a GEM of *S. suis* strain SC19 was constructed using ModelSEED (9) to address the limitations inherent in Tn-seq. Simulated gene deletions under defined conditions were used to predict whether the mutant strains could sustain viability. This approach identified 165 candidate essential genes (6), including 93 genes that were classified as non-essential by Tn-seq, but whose deletion abolished simulated growth (Fig. 1). Their apparent dispensability *in vivo* may reflect artifacts, such as metabolic compensation provided by complex media (whose nutritional profile is undefined and variable between batches), or the presence of suppressor mutations arising during the extended 12 hour post-transposition recovery phase. On the other hand, some genes deemed dispensable by GEM were determined to be essential in the Tn-seq data set. Aside from issues associated with Tn-seq and the sample recovery procedure discussed, the discrepancy may also likely be due to biological features not captured by the model, such as incomplete pathway annotation, a particular issue in works related to non-model species, including *S. suis*.

By integrating GEM and Tn-Seq results, Zhang et al. (6) identified a total of 244 candidate essential genes: 93 predicted only by GEM, 76 detected exclusively by Tn-seq, and 75 supported by both approaches (Fig. 1). This overlap not only illustrates the complementarity of the two methods but also highlights their respective biases. Genes validated by both strategies are likely to represent a core set of truly essential functions.

As anticipated, the final set includes established antibiotic targets, such as penicillin-binding proteins (β-lactam targets), as well as existing candidate pathways that feed into antibiotic discovery pipelines (e.g., lipid synthesis and cell division) (6). In addition, the study also identified several previously uncharacterized genes, as well as others involved in nucleoside sugar metabolism and signal transduction (6). Although the precise functions of many of these genes and how their disruption leads to cell death remain to be determined, these findings highlight the value of combining Tn-seq with GEM to dissect bacterial physiology and support antimicrobial target discovery.
